# Development of a Self-Help Smoking Cessation Intervention for Dual Users of Tobacco Cigarettes and E-Cigarettes

**DOI:** 10.3390/ijerph18052328

**Published:** 2021-02-27

**Authors:** Lauren R. Meltzer, Vani N. Simmons, Bárbara Piñeiro, David J. Drobes, Gwendolyn P. Quinn, Cathy D. Meade, Karen O. Brandon, Amanda Palmer, Marina Unrod, Paul T. Harrell, Christopher R. Bullen, Thomas Eissenberg, Thomas H. Brandon

**Affiliations:** 1 Department of Health Outcomes and Behavior, Moffitt Cancer Center, Tampa, FL 33612, USA; lauren.meltzer@moffitt.org (L.R.M.); vani.simmons@moffitt.org (V.N.S.); barpine82@gmail.com (B.P.); david.drobes@moffitt.org (D.J.D.); cathy.meade@moffitt.org (C.D.M.); karen.brandon@moffitt.org (K.O.B.); palmeram@musc.edu (A.P.); marinauf@yahoo.com (M.U.); 2 Department of Psychology, University of South Florida, Tampa, FL 33612, USA; 3 Department of Obstetrics and Gynecology and Population Health, School of Medicine, New York University, New York, NY 10016, USA; Gwendolyn.Quinn@nyulangone.org; 4 Department of Pediatrics, Eastern Virginia Medical School, Norfolk, VA 23501, USA; HarrelPT@EVMS.EDU; 5 Department of Population Health, University of Auckland, Auckland 1142, New Zealand; c.bullen@auckland.ac.nz; 6 Department of Psychology, Virginia Commonwealth University, Richmond, VA 23220, USA; teissenb@vcu.edu

**Keywords:** dual use, e-cigarette, self-help, smoking cessation, qualitative, focus groups, in-depth interviews

## Abstract

Most users of electronic cigarettes (e-cigarettes) report initiating use to quit combustible cigarettes. Nevertheless, high levels of dual use (i.e., using both combustible cigarettes and e-cigarettes) occur among adults. Using formative data from in-depth interviews and employing learner verification, we adapted an existing, validated self-help smoking-cessation intervention (Stop Smoking for Good; SSFG) to create a targeted intervention for dual users, If You Vape: A Guide to Quitting Smoking (IYV). In Phase I, in-depth interviews (*n* = 28) were conducted to assess relevance of the existing *SSFG* materials (10 booklets, nine pamphlets) and identify new content for the booklets. Next, for Phase II, learner verification interviews (*n* = 20 dual users) were conducted to assess their appeal and acceptability. Several key themes emerged from the Phase I in-depth interviews. Findings led to the inclusion of e-cigarette-specific strategies used by successful quitters such as gradually reducing nicotine levels, switching from tobacco flavor to alternative flavors, and limiting e-cigarette use to places one would normally smoke (i.e., not expanding use). Suggestions from Phase II learner verification included broadening the visual appeal for a younger, more diverse demographic, expanding tips for quitting smoking via e-cigarettes, and expanding terminology for e-cigarette devices. Beginning with an efficacious self-help intervention, we used a systematic process to develop a version specifically for dual users.

## 1. Introduction

Electronic cigarettes (e-cigarettes) have been on the market for over a decade, and evidence of their efficacy for smoking cessation is emerging [1,2,3]. Although the majority of e-cigarette users (vapers) report initiating use to quit or reduce smoking combustible cigarettes [4,5], high levels of dual use (i.e., using both combustible cigarettes and e-cigarettes) occur among adults. For example, in 2016, 54.6% of current US e-cigarette users were also current cigarette smokers [6]. Because dual users typically receive nicotine from both combustible cigarettes and e-cigarettes, there is concern that e-cigarettes may maintain or increase nicotine dependence [7] and may, therefore, facilitate continued smoking among those otherwise motivated to quit.

It is possible that, if they deliver nicotine effectively to the user, e-cigarettes might function similarly to traditional FDA-approved nicotine replacement therapies (NRT). Qualitative research suggests that smokers find e-cigarettes more appealing than conventional NRT because of perceived greater biobehavioral similarity to cigarette smoking, better reduction in cravings, and fewer side effects [8,9]. Moreover, in a survey of vapers comparing e-cigarettes to NRT, e-cigarettes were rated more useful for quitting smoking, better at reducing tobacco cravings, less addictive, more convenient, and less expensive [4]. Survey respondents also rated e-cigarettes as being less hazardous to their health as compared to NRT. Although millions of smokers are simultaneously using e-cigarettes, often with the aim of quitting smoking, they usually do so without receiving any behavioral assistance. Such dual users are receiving nicotine from both products, and although greater use of nicotine per se is not particularly troublesome (i.e., use of NRT can be recommended to patients who are actively smoking), there are concerns that dual using may not only hinder the quitting process for combustible cigarettes but may increase health risks due to exposure to vaping-related toxins. There is, thus, a need for additional assistance to enhance the chance of achieving tobacco abstinence. Previous research shows the efficacy of NRT is improved when combined with even minimal behavioral interventions [10,11]. Thus, an opportunity exists to assist the large population of self-selected and motivated smokers who have already demonstrated a potential first step toward smoking cessation by commencing use of e-cigarettes, using a minimal behavioral intervention to facilitate sustained smoking abstinence. 

Our research team previously developed a self-help smoking cessation intervention (titled Stop Smoking for Good; SSFG) that significantly enhanced cessation in a general sample of smokers over 18 months of follow-up compared to control (33 vs. 23% smoking abstinence, respectively) [12]. SSFG contains 10 booklets covering various topics related to smoking cessation (e.g., mood, health, weight gain) as well as nine individual pamphlets with testimonials that act as social support. Key characteristics that distinguish this self-help intervention from others with limited efficacy include content based on both cognitive-behavioral theory and empirical research, and the intervention extends (via multiple booklets) over time, through 18 months. Given the evidence showing that dual users are generally motivated to quit smoking, dual use often persists, e-cigarettes can aid smoking cessation, and behavioral factors contribute to the efficacy of e-cigarettes for smoking cessation, we sought to adapt the SSFG intervention for dual users of combustible cigarettes and e-cigarettes. With this potential, the goal of the current study was to develop the first self-help smoking cessation intervention specifically for dual users of combustible cigarettes and e-cigarettes based on the validated SSFG smoking-cessation self-help intervention [12]. The aim was to create an intervention adapted to the unique needs, circumstances, and risk factors of dual users that would provide assistance for smoking cessation and, if desired, eventual cessation of e-cigarettes as well. Thus, qualitative research methodologies were ideal to gain an understanding of dual users’ perspectives and experiences. Importantly, the availability of such an intervention represents a novel approach by focusing on individuals who have already initiated vaping and teaching them to use e-cigarettes as an aid to smoking cessation rather than as a product that may contribute to the maintenance of smoking. This paper describes the series of iterative steps leading to the development of this targeted intervention, which would then be tested in a randomized controlled trial (RCT) [13].

## 2. Materials and Methods 

### 2.1. Intervention Development: Overview

A multi-phase approach was employed utilizing two complementary qualitative formative research methodologies—in-depth interviews and learner verification—to aid in the development of the intervention. All study phases were approved by the Liberty Institutional Review Board and occurred between May 2015 and May 2016.

### 2.2. Phase I: Individual in-Depth Interviews

We first conducted individual interviews to identify and explore new content topics for inclusion in the novel intervention and to gather feedback about the existing SSFG booklets in terms of tone, visual elements, and other important fundamentals of smoking cessation message design for this audience. 

#### 2.2.1. Participant Recruitment and Data Collection

Inclusion criteria were ≥18 years of age; able to speak and read English; ≥1 year history of daily smoking; ≥1 month of e-cigarette use. Participants were recruited in Florida via multimedia advertisements (press releases, newspaper advertisements, e-cigarette forums, and flyers). Interested participants were screened by study staff, and eligible participants were categorized into one of 4 pre-determined subgroups based on their unique perspectives to inform a future intervention for dual users: (1) current dual users without interest in quitting smoking (hereafter referred to as unmotivated dual users); (2) current dual users who had attempted but had not quit smoking; (3) current e-cigarette users who had successfully quit smoking; (4) former dual users who had quit both products. These subsets represent the continuum from dual use through successful cessation of both products, allowing us to learn from individuals with a wide range of smoking and e-cigarette experiences and perspectives. Participants provided written informed consent and, then, completed an in-person interview with a study investigator trained in qualitative interviewing (V.S. or G.Q.). Saturation was achieved (i.e., no new themes emerged) after completion of 28 interviews, as determined by interim analysis that involved de-briefing between interviewers after each interview [14]. The interviews took approximately 1 h to complete and participants received 30 USD cash for their time. 

#### 2.2.2. Measures and Data Analysis

A semi-structured interview guide was used to ask participants a series of open-ended questions to identify perspectives about the benefits of quitting smoking, barriers to quitting smoking, and how e-cigarettes help or hinder the quitting process. In addition, all participants were asked to review and provide feedback on the existing 10 *SSFG* booklets and 9 pamphlets. The first booklet provides a general overview about quitting smoking, and each of the remaining 9 booklets includes more extensive information on a topic related to quitting smoking such as nicotine dependence, behavioral and cognitive coping strategies for dealing with urges to smoke, stages of quitting, smoking and weight, and how to cope with stress and negative moods without smoking. In addition to the 10 booklets, 9 tri-fold color pamphlets (A Forever Free Close-Up: How I Quit Smoking) reinforce key messages about quitting smoking (e.g., dealing with stress, finding other forms of positive reinforcement). To induce a sense of social support, the messages are communicated as a first-person narrative from a former smoker. 

Table 1 lists example questions from the interview guide. Following the interview, participants completed a brief, self-report questionnaire assessing the history of combustible cigarette and e-cigarette use and dependence [15] as well as demographic and tobacco history including the Fagerström Test for Nicotine Dependence (FTND) [16]. These measures were included to describe the sample with respect to sociodemographic, smoking, and vaping characteristics. All interviews were audio-recorded and professionally transcribed verbatim. Once saturation was reached, systematic data analysis was conducted. Inductive content analysis and the constant comparison method were used to guide data analysis [17]. Three members of the team conducted the coding and analysis. First, using the semi-structured interview guide, open coding was used to develop an initial code book with code names, meanings, and examples. Then, the coders reviewed all of the transcripts, applied the codes, and held discussions to identify emergent (new) themes and update the codebook. Differences in coding were resolved via discussion amongst the team until consensus was reached. The inter-rater reliability rate was 81% between all coders with the final codebook.

### 2.3. Phase II: Learner Verification Interviews

Based on the results of the Phase I interviews, as well as the emerging literature on e-cigarettes and smoking cessation, the *SSFG* materials were modified into the first draft of the new intervention, titled If You Vape: A Guide to Quitting Smoking (IYV). During Phase II, learner verification interviews were conducted on the IYV draft with participants who fit inclusion criteria for the planned RCT: (1) ≥18 years of age; (2) smoking ≥1 combustible cigarette/week over the past year; (3) e-cigarette use ≥1/week over the past month; (4) not currently enrolled in a face-to-face smoking cessation program; (5) able to speak and read English. The purpose of learner verification is to enhance the suitability of the materials by assessing acceptability, attraction, understanding, self-efficacy, and persuasion [18,19]. We utilized two rounds of interviews during this phase. Following the first set, feedback was incorporated into the booklets, and the revised booklets were used for the second round of participant interviews. Given that only small samples are needed for learner verification [18,19], 10 participants were recruited for each round. Participants provided written informed consent and completed a self-report questionnaire mirroring that of the baseline questionnaire for the planned RCT. The survey contained items assessing demographic characteristics, as well as combustible cigarette use history and e-cigarette use. All baseline measures are described elsewhere [13]. The interviews took approximately 1 h to complete, and participants received USD 30 cash for their time. 

Using a semi-structured interview guide, participants were asked to evaluate key elements of the new intervention content with respect to appeal, comprehension, and persuasion. We also assessed if dual users found the materials to be relatable and acceptable, and we evaluated the new e-cigarette-specific content in the booklets. To do so, participants were shown examples and specific sections of the booklets that corresponded to each of the aspects above. Examples of questions included: “What do you think about the length of the booklets?”; “We want to be sure people who read these booklets know they are for dual users. Is this clear by looking at the booklet covers?”; “Throughout the booklets we refer to e-cigarettes and their products in a variety of ways. We use terms such as e-cigarette, vape pen, cigalike, vaping, e-juice, etc. Do you feel these are acceptable terms to use? Are there any others that might be more clear?”; “Do you feel the suggestions given in these booklets are realistic and doable? Could you do them?”; “Please read the ‘Frequently asked questions about using e-cigarettes to quit smoking’ section. Are these questions helpful? Are there any other questions that you want answered or think we should include?” Interviews were audio recorded, and data were tabulated through descriptive summaries and data display matrices to determine modifications and revisions for the next draft. Members of the research team reviewed, validated, and verified interpretations. Data collected during the first half of interviews informed additional modifications to the original 9 *SSFG* supportive pamphlets. All modifications were reviewed by the research team and agreed upon through consensus. The second round of interviews was used to verify the modified content was acceptable/appealing, understandable, and persuasive. Feedback from the participant interviews and the research team was incorporated to produce the final intervention, resulting in a refreshed, targeted series of booklets named If You Vape: A Guide to Quitting Smoking (IYV).

## 3. Results

### 3.1. Participant Characteristics

Characteristics of in-depth interview participants (Phase I; *n* = 28) and learner verification participants (Phase II; *n* = 20) are presented in Table 2. Approximately, one-half of in-depth interview participants were male (54%) with a mean age of 48.8 years (SD = 14.2; range 19–72). The majority were Caucasian, almost half were married, and the majority had some college education or beyond. The majority of the in-depth interview participants reported an income of less than USD 30,000 a year. Of the learner verification participants (*n* = 20), most were male (70%) and Caucasian (75%), with a mean age of 42.5 years (SD = 14.5), and they smoked a median of 16–20 CPD, with low nicotine dependence as indicated by the FTND.

### 3.2. Phase I in-Depth Interviews: Key Findings

Key themes and quotes from the in-depth interviews and feedback pertaining to the *SSFG* intervention are presented below. 

#### 3.2.1. Reasons for Initiating E-Cigarette Use

Participants who had already quit smoking or had an interest in quitting smoking stated that the primary reason for initiating e-cigarette use was to quit smoking. In contrast, unmotivated dual users noted other reasons for initiating e-cigarette use (e.g., the desire to vape in places where smoking was prohibited, out of curiosity, to try the flavors, etc.).

“*I know it’s an issue in public areas, using regular tobacco because people don’t want you to use regular tobacco in public areas. So I kind of tried to use that—or in the car or something, so it doesn’t smell. People don’t mind it as much.*”

—Unmotivated dual user.

“*I had smoked for 48 years. I got sick, I had never been sick like this… I went to my primary care physician…Anyway, I got sick, I got scared, and she recommended the e-cigarettes. I did it cold turkey with the e-cigarettes, and I haven’t smoked since.*”

—Former dual user who had successfully quit both products.

#### 3.2.2. Positives and Negatives of E-Cigarettes

The majority of responses were similar across all four groups. Participants frequently mentioned that e-cigarettes were healthier, less expensive, less stigmatizing, and better smelling as compared to combustible cigarettes. The primary negative characteristics mentioned by most participants included not knowing enough about the chemicals in the devices and e-liquids and that e-cigarettes can require a lot of maintenance and upkeep. Unmotivated dual users found the ability to use e-cigarettes continuously and anywhere to be a positive characteristic, whereas those who had successfully quit smoking believed this to be a negative because it led to increased smoking and increased nicotine dependence.

“*Well, certainly it’s something you can smoke almost anywhere. I have on occasion taken it out to when I went out for dinner and snuck a few puffs in a restaurant…Or, just in my own house when I have guests and they’re non-smoking I’m able to smoke that without aggravating them.*”

—Unmotivated dual user.

“*Yeah, I was using it more frequently, right. I had it in my hand and mouth a lot more than I did the [tobacco cigarettes]… and there’s no end to it. It’s not like there’s a cigarette with a definitive end… I mean, if you’re trying to stop smoking then it’s not a positive, it’s a negative…*”

—Former dual user who had successfully quit both products.

#### 3.2.3. NRT Compared to E-Cigarettes

The majority of participants indicated previous use of one or more forms of traditional NRT. Most respondents in all four groups agreed that e-cigarettes were better than traditional NRT for quitting smoking.

“*I think e-cigarettes are better than those [NRT] because you’re not completely cold turkey replacing. You’re having something to substitute that sensation of having a cigarette in your hand.*”

—Current dual user who has not been able to quit smoking.

“*They far surpass it… I was a pack-and-a-half-a-day smoker for 19 years. Of all the methods that I tried, I was never able to quit longer than 30 days. In this instance, I quit immediately and I have not had any desire to smoke a cigarette over the course of the entire use of the electronic cigarette.*”

—E-cigarette user that successfully quit smoking.

#### 3.2.4. Perceived Advantages, Disadvantages, and Reasons for Continued Dual Use 

Unmotivated dual users were asked to describe the advantages and disadvantages of dual use. A few noted that combustible cigarettes were better in times of stress and that they provide a better nicotine “hit”. One participant stated that combustible cigarettes were more readily available for purchase than e-cigarette devices and e-liquid, whereas another indicated that e-cigarettes were good to have when in places where smoking is prohibited. Almost all participants believed there were no negatives to dual use.

“*I think it’s psychological, for me…Like if I get upset, or like with coffee, it’s a habit…But when I get upset, it’s more of a comfort thing…I can get the nicotine through the e-cigarette, but it’s more of a connection of something that—I’m battling with whether or not I really wanna let go.*”

—Current dual user who has not been able to quit smoking.

The majority of dual users believed the ability to quit smoking was psychological, in essence, or that smoking was a habit they were unable to break. When successful quitters were asked to speculate on what barriers dual users had that prevented smoking cessation, they stated that they most likely had not yet found the right e-cigarette device, flavor, or nicotine level.

“*I maybe attribute it that they’re not using the correct device for them or they’re not using the correct e-liquid, the right milligram.*”

—E-cigarette user that successfully quit smoking.

“*Either the nicotine is too low or too high. The coil is wrong for that person. The flavor of the juice could be wrong for that person. It could be a lot of factors.*”

—E-cigarette user that successfully quit smoking.

#### 3.2.5. Recommendations for Using E-Cigarettes to Quit Smoking 

Participants who had successfully quit smoking were asked if they would recommend e-cigarettes to someone trying to quit smoking, and what suggestions they would provide on how to use an e-cigarette to quit. Almost all participants said they would recommend e-cigarettes for smoking cessation. One participant suggested vaping only in response to cravings to smoke a combustible cigarette. Another participant recommended initially using an e-liquid with similar nicotine delivery as one’s combustible cigarettes to help with the transition. Another participant advised vaping only in places where one would normally smoke combustible cigarettes (i.e., do not increase range of use). A few others suggested to lower the nicotine level over time and to change the flavors away from tobacco or menthol.

#### 3.2.6. Stop Smoking for Good Intervention Feedback 

Participants were asked to provide feedback on the *SSFG* booklets with regards to the specific smoking cessation content relevant to dual users. First, the majority of participants felt that e-cigarettes should not be listed along with traditional forms of NRT if they were not FDA-approved. Participants read a section discussing nicotine addiction, and the majority of participants said they felt the information only pertained to combustible cigarettes and not e-cigarettes. Additional feedback included participants’ willingness to use NRT in situations when they were unable to vape, the need to emphasize that e-cigarettes have been helpful for coping with withdrawal symptoms when quitting smoking, and that e-cigarettes can also be just as helpful as combustible cigarettes for alleviating stress.

### 3.3. Summary of Changes Made to Materials Based on in-Depth Interviews

Given that not all e-cigarette users had considered e-cigarettes as a smoking cessation tool, we developed a brief introductory brochure to introduce the IYV booklets and to describe the goal of harnessing e-cigarette use as a tool to assist with smoking cessation. Based on suggestions from successful quitters, the existing *SSFG* booklets were modified to address e-cigarette-specific strategies for quitting smoking, including gradually reducing nicotine levels, switching from tobacco/menthol flavor to an alternative flavor, and limiting e-cigarette use to places one would normally use combustible cigarettes. Additionally, vignettes within the booklets were modified to address topics such as using e-cigarettes to reduce nicotine withdrawal symptoms and craving to smoke, as well as to cope with stress. We also emphasized the importance of finding the right device and e-liquid (flavor and nicotine content). Additional content was added to raise awareness of the potential for nicotine dependence from e-cigarettes. Finally, the later booklets introduced the idea of eventually quitting e-cigarettes once the reader had successfully quit smoking. The supportive pamphlets were also rewritten to include the perspectives of e-cigarette users who successfully quit smoking while reinforcing and illustrating the key points from the booklets.

### 3.4. Learner Verification: Key Findings

Findings from the learner verification interviews resulted in a number of revisions and modifications to the booklets and pamphlets. Findings and changes are listed below.

#### 3.4.1. Visual Appeal of the IYV Booklets and Pamphlets

Overall, participants found the visual appearance of the booklets and pamphlets engaging. They reported that the titles fit the content of the booklets and made them want to see what was inside. However, when looking at the covers, participants felt it was not obvious that the booklets were for dual users. Based on this feedback, different photos were selected for the covers to include more e-cigarette-related images. Images inside the materials were also evaluated and found to be acceptable to the majority of participants. However, participants felt that some of the images looked staged or that the devices in the photos appeared dated. Those photos were all replaced with new, contemporary images that were selected and approved by study participants. 

#### 3.4.2. Comprehension of Messages 

Overall, participants understood that the materials were designed to assist individuals with quitting smoking using their e-cigarette. When asked about the e-cigarette-specific terms for users, products, and devices, most participants found the terms acceptable. However, a number of participants were unfamiliar with the term “cigalike,” so it was removed from the materials.

#### 3.4.3. Relatability and Acceptability of Booklets and Pamphlets 

Participants felt that the personal stories in the booklets and pamphlets were relatable and easy to understand. Multiple participants expressed that it was “nice to have someone to relate to.” One participant, however, felt that he was able to relate to the content of the personal stories, but not the images. As a result, all of the personal story images were reviewed, and new images were selected to ensure relatability across a range of demographic groups. The new images were received positively during the second round of interviews.

#### 3.4.4. Persuasiveness of Content

The vast majority of participants, in both rounds of interviews, believed the materials would both encourage and motivate dual users to quit smoking using their e-cigarette. Some participants also expressed an increased desire to quit smoking themselves after reading the materials and a few asked to take a set of the booklets home. 

#### 3.4.5. Evaluation of New E-Cigarette-Specific Content in the Booklets

In the revised introductory overview booklet, participants found the newly developed “Frequently asked questions about using e-cigarettes to quit smoking” and “Using e-cigarettes to quit smoking” sections to be helpful, realistic, and feasible. Keeping an extra battery on hand was provided as an additional suggestion. Participants were asked about a section of Booklet 5 (Your Health) that discusses the dangers of cigarettes and e-cigarettes. All participants reported that, based on the booklet content, they believed e-cigarettes were less harmful than combustible cigarettes. Content pertaining to quitting e-cigarettes first appeared in Booklet 8 (Life without Cigarettes), and participants felt the approach and timing of these messages were appropriate and not off-putting. To complement an existing table displaying the amount saved by not smoking combustible cigarettes, an additional table was included in Booklet 8 to convey the amount spent per week on vaping supplies. The majority of participants liked this addition and found it to be beneficial. Booklet 9 (Benefits of Quitting Smoking) included the benefits from quitting e-cigarettes. All participants reported that this information was helpful, appealing, and motivating for quitting e-cigarettes. Table 3 provides an overview of the IYV intervention materials, as well as the distribution schedule for the RCT.

## 4. Discussion

This new intervention provides assistance for smoking cessation tailored for e-cigarette users and encourages eventual cessation of e-cigarettes. Our development process was comprehensive, using a multi-phase, qualitative approach, including in-depth interviews and learner verification to ensure that the content, format, and overall approach was consistent with the needs of the intended audience. We drew upon existing literature, our own prior smoking cessation, and e-cigarette research [5,12] and integrated the findings from the in-depth interviews and learner verification interviews. Findings from our formative research were used to modify the *SSFG* series, including the addition of an introductory brochure to explain the objectives of the IYV series. Other modifications included the addition of contemporary images of a range of e-cigarette devices and diverse user populations. Feedback from successful quitters led to the addition of content describing e-cigarette-specific strategies for quitting smoking, including commencing with a tobacco flavored e-liquid then switching to an alternative flavor, gradually reducing the nicotine content, and only using an e-cigarette in places where one would typically smoke combustible cigarettes. Additionally, current dual users reported a number of barriers to quitting smoking, such as coping with stress, nicotine withdrawal, and difficulties selecting the right device or e-liquid. The vignettes in the booklets and pamphlets were modified to address these topics. Finally, special targeted sections were created to address misconceptions about the harms of nicotine and to discuss the benefits of quitting e-cigarettes. 

A limitation of this research is that all data were collected in just one jurisdiction (Florida, USA). Perspectives may differ based on differing messaging and regulations regarding e-cigarettes across states and nations. In addition, our research preceded a major evolution of e-cigarette technology, specifically the advent of “pod mod” devices, such as JUUL^®^, that use protonated nicotine and have since gained market dominance over tank systems. However, it is likely that the fundamental content of our materials will continue to apply to the newer technology, albeit with the need for regular updates for relevance and device-specific advice. Additionally, public policy and regulation of e-cigarettes and other nicotine delivery systems have continued to evolve, which impact both product availability and public perceptions. Changing perceptions may, in turn, affect smokers’ consideration of e-cigarettes for smoking cessation and require additional clarification of potential health risks and benefits within the intervention materials. Finally, future interventions will need to consider the role of flavors in potentially facilitating smoking cessation given recent efforts to ban flavored e-cigarettes. 

## 5. Conclusions

Our systematic approach to intervention development informed several key changes to our materials. For example, data collected from Phase I in-depth interviews led to the addition of e-cigarette-specific strategies used by successful quitters. Suggestions from Phase II learner verification interviews resulted in broadening the visual appeal for a younger, more diverse demographic, expanding tips for quitting smoking via e-cigarettes, and expanding terminology for e-cigarette devices. We followed the development of these intervention materials with an RCT, in which we compared this IYV intervention with general self-help materials that were not adapted specifically for dual users, and with an assessment-only control condition [13]. Self-help interventions such as this have the advantage of relatively easy dissemination and wide reach. Thus, pending the results of the RCT, future efforts will focus on dissemination and implementation.

## Figures and Tables

**Table 1:** In-depth interview guide sample questions.

**All Groups**
Tell me about your experience with starting e-cigarettes.
2. How do you refer to your device? Would you be offended if someone referred to it as an e-cigarette?
3. Tell me about some of the positives or negatives of using e-cigarettes? How do e-cigarettes compare to regular tobacco cigarettes? Have you used NRT? How do e-cigarettes compare to nicotine replacement therapies?
4. What do you think are the benefits of using both e-cigarettes and traditional cigarettes? What do regular cigarettes provide that e-cigarettes don’t?
5. Are there negative aspects to using both?
6. Do you think using an e-cigarette makes it easier or harder to quit smoking?
7. Would you be interested in receiving smoking cessation materials?
8. Would you be interested in receiving information and help about using e-cigarettes to quit smoking?
**Stop Smoking for Good booklets**
Do you feel information about quitting e-cigarettes should come early on in the booklet series or towards the end?
2. Do you view e-cigarettes as a quit smoking aid like NRTs? Do you feel e-cigarettes should be included when talking about NRT’s, or should they be discussed separately?
**Current Dual Users—No Interest in Quitting & Attempted but not quit smoking**
Why do you think e-cigarettes have not been enough to help you quit smoking?
**Current e-cigarette users who have quit smoking**
Why do you think e-cigarettes helped you to successfully quit smoking tobacco cigarettes?
**Former dual users who quit both e-cigarettes and traditional cigarettes**
How did you quit smoking? How, specifically, did e-cigarettes play a part/help you?
2. What suggestions would you give for how to use an e-cigarette to help you quit smoking?

**Table 2:** In-depth interviews and learner verification: participant demographic and smoking characteristics.

	Phase I: In-Depth Interviews					Phase II: Learner Verification
	Total *n* = 28	Dual Users w/o Interest in Quitting Smoking *n* = 8	Dual Users Who Attempted to Quit Smoking *n* = 8	E-Cigarette Users Who Quit Smoking *n* = 7	Former Dual Users Who Have Quit Both Products *n* = 5	Total *n* = 20
Participant Characteristics—M (SD) or N%						
Gender (Male)	15 (54%)	6 (75%)	5 (63%)	2 (29%)	2 (40%)	14 (70%)
Age	48.8 (14.2)	36.6 (16.2)	52.4 (9.5)	49.3 (8.9)	62.0 (9.3)	42.5 (14.5)
Race: Caucasian	23 (82%)	6 (75%)	7 (88%)	5 (71%)	5 (100%)	15 (75%)
Marital Status: Married	12 (43%)	2 (25%)	6 (75%)	3 (43%)	1 (20%)	3 (15%)
Education: Some college or beyond ^2^	22 (79%)	7 (88%)	6 (75%)	5 (72%)	4 (80%)	13 (65%)
Income: ≤30,000 ^3^	20 (74%)	6 (75%)	6 (75%)	5 (71%)	3 (75%)	6 (30%)
Smoking and Vaping Characteristics—M (SD) or *N*%						
CPD (Median) ^1^	16–20	6–10	16–20	16–20	16–20	16–20
Years smoked ^1^	31.5 (15.0)	19.1 (15.6)	37.3 (11.0)	30.0 (8.9)	44.0 (13.6)	19.3 (12.4)
Fagerström Test for Nicotine Dependence (FTND) ^1^	3.7 (1.7)	3.1 (1.7)	4.83 (1.8)	3.1 (1.0)	4.2 (1.8)	3.4 (2.0)
Months vaping: ≥1 year	19 (70%)	5 (63%)	4 (50%)	7 (100%)	3 (75%)	12 (60%)
Vaping Frequency: 7 days/week ^2^	20 (71%)	4 (50%)	6 (75%)	7 (100%)	3 (60%)	12 (60%)

^1^ Smoking characteristics reflected current usage for the first two groups (current smokers) and previous highest usage for the last two groups (former smokers). ^2^ Education not reported for 2 participants. ^3^ Income not reported for 1 participant.

**Table 3:** Final intervention booklets and pamphlets: titles, themes, and distribution schedule.

Intervention Materials	Theme	Month Sent
Introductory Brochure	If You Vape: About this Book Series	0
Booklet 1	If You Vape: A Guide to Quitting Smoking: An Overview	0
Booklet 2	Smoking Urges	1
Booklet 3	Smoking and Weight	2
Booklet 4	What if you have a cigarette?	3
Pamphlet 1: Anna’s Story	Quitting smoking	4
Booklet 5	Your Health	5
Pamphlet 2: Joe’s Story	Pharmacotherapy	6
Booklet 6	Smoking, Stress and Mood	7
Pamphlet 3: Chen’s Story	Smoking urges	8
Booklet 7	Lifestyle Balance	9
Pamphlet 4: Gloria’s Story	Managing a slip	10
Pamphlet 5: Candice’s Story	A healthier life	11
Booklet 8	Life without cigarettes	12
Pamphlet 6: Maria’s Story	Weight gain concerns	13
Pamphlet 7: Steve’s Story	Social support	14
Booklet 9	The benefits of quitting smoking	15
Pamphlet 8: Carlos’s Story	Remaining smoke free among other smokers	16
Pamphlet 9: Warren’s Story	Positive addictions	17
Booklet 10	The Road Ahead	18

## Data Availability

The qualitative data used in this study are not available due to privacy restrictions.
